# Immunotherapy response and microenvironment provide biomarkers of immunotherapy options for patients with lung adenocarcinoma

**DOI:** 10.3389/fgene.2022.1047435

**Published:** 2022-10-25

**Authors:** Xue Zhan, Shihan Feng, Xutao Zhou, Wei Liao, Bin Zhao, Qian Yang, Qi Tan, Jian Shen

**Affiliations:** ^1^ Chongqing Key Laboratory of Traditional Chinese Medicine for Prevention and Cure of Metabolic Diseases, College of Traditional Chinese Medicine, Chongqing Medical University, Chongqing, China; ^2^ Department of Oncology, Jiulongpo Hospital of Traditional Chinese Medicine, Chongqing, China; ^3^ Department of Oncology, Chongqing Hospital of Traditional Chinese Medicine, Chongqing, China

**Keywords:** lung adenocarcinoma, immunotherapy response, ERVH48-1, tumor environment, predictive model

## Abstract

**Background:** Immunotherapy has been a promising approach option for lung cancer.

**Method:** All the open-accessed data was obtained from the Cancer Genome Atlas (TCGA) database. All the analysis was conducted using the R software analysis.

**Results:** Firstly, the genes differentially expressed in lung cancer immunotherapy responders and non-responders were identified. Then, the lung adenocarcinoma immunotherapy-related genes were determined by LASSO logistic regression and SVM-RFE, respectively. A total of 18 immunotherapy response-related genes were included in our investigation. Subsequently, we constructed the logistics score model. Patients with high logistics score had a better clinical effect on immunotherapy, with 63.2% of patients responding to immunotherapy, while only 12.1% of patients in the low logistics score group responded to immunotherapy. Moreover, we found that pathways related to immunotherapy were mainly enriched in metabolic pathways such as fatty acid metabolism, bile acid metabolism, oxidative phosphorylation, and carcinogenic pathways such as KRAS signaling. Logistics score was positively correlated with NK cells activated, Mast cells resting, Monocytes, Macrophages M2, dendritic cells resting, dendritic cells activated and eosinophils, while was negatively related to Tregs, macrophages M0, macrophages M1, and mast cells activated. In addition, ERVH48-1 was screened for single-cell exploration. The expression of ERVH48-1 increased in patients with distant metastasis, and ERVH48-1 was associated with pathways such as pancreas beta cells, spermatogenesis, G2M checkpoints and KRAS signaling. The result of quantitative real-time PCR showed that ERVH48-1 was upregulated in lung cancer cells.

**Conclusion:** Our study developed an effective signature to predict the immunotherapy response of lung cancer patients.

## Introduction

Deaths from lung cancer account for a significant percentage of all cancer-related deaths (Nguyen et al., 2022). There are many types of lung cancer, but the majority of them are non-small cell lung cancer (NSCLC) (Zhang et al., 2021). As cancer genomics has advanced in the past few decades, several mutations have been identified as lung adenocarcinoma (LUAD) driver genes, such as KRAS, epidermal growth factor receptors (EGFRs), c-METs, and so on (Chen et al., 2019; Wang et al., 2019; Sun et al., 2020; Wang et al., 2020). A variety of drugs have since been developed to target mutations in driver genes. Despite receiving targeted therapies, most patients eventually develop resistance to them, partly due to secondary mutations in the tumor (Foggetti et al., 2021; Tumbrink et al., 2021).

Biologically-based cancer immunotherapy provides new perspectives for cancer treatment (Denisenko et al., 2018). Generally, LUAD often has high tumor mutational burden (TMB) and immunogenicity characteristics (Skoulidis et al., 2018). Therefore, LUAD is an ideal immunotherapy indication (Zhang et al., 2020). Nowadays, immune therapy, like immune checkpoints (ICI), has shown encouraging results. ICI treatment, however, is relatively ineffective for LUAD patients (Santarpia et al., 2020). So far, a series of biomarkers that predict the treatment effectiveness of ICI have been verified, including immune cell status, PD-L1 expression levels, neoantigens, intestinal flora, and TMB (Peng et al., 2020). Transcriptome sequencing files have recently made it possible to estimate the immune status of cancer using the Tumor Immune Dysfunction and Exclusion (TIDE) algorithm. The core is to determine whether T cells are depleted in immune “hot” tumors, or whether there are three types of suppressive T cell infiltration in immune “cold “tumors (Connolly et al., 2021; Shao et al., 2021). There is an urgent need to build a model based on immunotherapy-related genes to indicate the clinical efficacy of ICI.

Cancer immune status is closely related to the prognosis of patients and can indicate the effect of immunotherapy (Ren et al., 2021; Yu et al., 2021). In our investigation, the lung cancer patients were divided into the corresponding immunotherapy responders and non-responders groups through the TIDE algorithm. The characteristic genes of immunotherapy were screened by LASSO logistic regression and the SVM-RFE algorithm. Logistics regression analysis was used to establish a logistics model based on the immunotherapy characteristic genes, and each patient was assigned a logistics score according to the identified formula. Furthermore, we investigated the underlying difference in patients with high and low logistic score. Finally, ERVH48-1 was identified as an underlying target to interfere with the response of immunotherapy in LUAD patients.

## Methods

### Data acquisition

The transcriptomic data and clinical information of lung adenocarcinoma patients were obtained from the Cancer Genome Atlas database (TCGA-LUAD project). The format of transcriptomic data was STAR-counts and the format of clinical data was bcr-xml. All raw data was organized using the author’s R and Perl code. Before analysis, all the data were preprocessed, including probe annotation, missing value completion and Data standardization. The baseline information of enrolled patients was shown in Table 1.

**TABLE 1:** The baseline information of the TCGA-LUAD patients.

Clinical variable		Number	Percentage (%)
Age	≤65	241	46.2
	>65	262	50.2
	Unknown	19	3.6
Gender	Female	280	53.6
	Male	242	46.4
Stage	Stage I	279	53.4
	Stage II	124	23.8
	Stage III	85	16.3
	Stage IV	26	4.9
	Unknown	8	1.5
Tstage	T1	172	32.9
	T2	281	53.8
	T3	47	9.0
	T4	19	3.6
	Unknown	3	0.6
Mstage	M0	353	67.6
	M1	25	4.8
	Unknown	144	27.6
Nstage	N0	335	64.2
	N1	98	18.8
	N2	75	14.4
	N3	2	0.4
	Unknown	12	2.3

### Evaluation of immunotherapy response

Each patient’s response rate to immunotherapy was assessed using the Tumor Immune Dysfunction and Exclusion (TIDE) algorithm (Fu et al., 2020). Specifically, the cancer type was selected as NSCLC. Among them, patients with TIDE >0 were considered non-responders to immunotherapy, and patients with TIDE <0 were considered responders to immunotherapy.

### Machine learning algorithm and logistic model

The LASSO logistic regression and SVM-RFE algorithm were performed to optimize variable selection (Deo, 2015). The immunotherapy characteristic genes of LUAD were screened by LASSO logistic regression and SVM-RFE algorism. Logistics regression analysis was used to establish a logistics model based on the immunotherapy characteristic genes, and each patient was assigned a logistics score according to the identified formula.

### Biological enrichment

The Gene Set Enrichment Analysis (GSEA) algorithm was utilized to explore the differences in biological pathways (Subramanian et al., 2005). The reference gene set was Hallmark and c2. cp.kegg.v2022.1. Hs.symbols.gmt file. Gene Ontology (GO) analysis was conducted using the “Clusterprofiler” package (Yu et al., 2012).

### Immunocytes infiltration assessment

Immunocyte infiltration was quantified using the “CIBERSORT” algorithm (Chen et al., 2018).

### Genome instability analysis

TMB and microsatellite instability (MSI) were obtained directly from the TCGA database. Tumor stemness index mRNAsi and EREG-mRNAsi were obtained from previously published articles (Malta et al., 2018).

### Single-cell RNA-seq analysis

ERVH48-1 analysis at single-cell RNA-seq level was performed *via* the http://tisch.comp-genomics.org/home/website.

### Cell lines and quantitative real-time (qRT) PCR

Normal BEAS-2B and lung cancer A549, H1299, and SPC-A1 cell lines were laboratory stocks. Total RNA was extracted using an RNA extraction kit following the protocol. SyBr Green PCR system was used for the qRT-PCR. The primers used were as follows: ERVH48-1, forward primer, 5′-CTC​CGG​GTT​CCA​ACC​AAT​G-3′, reverse primer, 5′-AGA​GGC​GAC​TAG​AGG​CTG​AG-3’; GAPDH, forward primer, 5′- GGA​GCG​AGA​TCC​CTC​CAA​AAT-3′, reverse primer, 5′- GGC​TGT​TGT​CAT​ACT​TCT​CAT​GG-3’.

### Statistical analysis

An analysis of the data was conducted using R software, and a *p* value of 0.05 on both sides was considered statistically significant.

## Results

### Screening immune response-related genes

**FIGURE 1:**
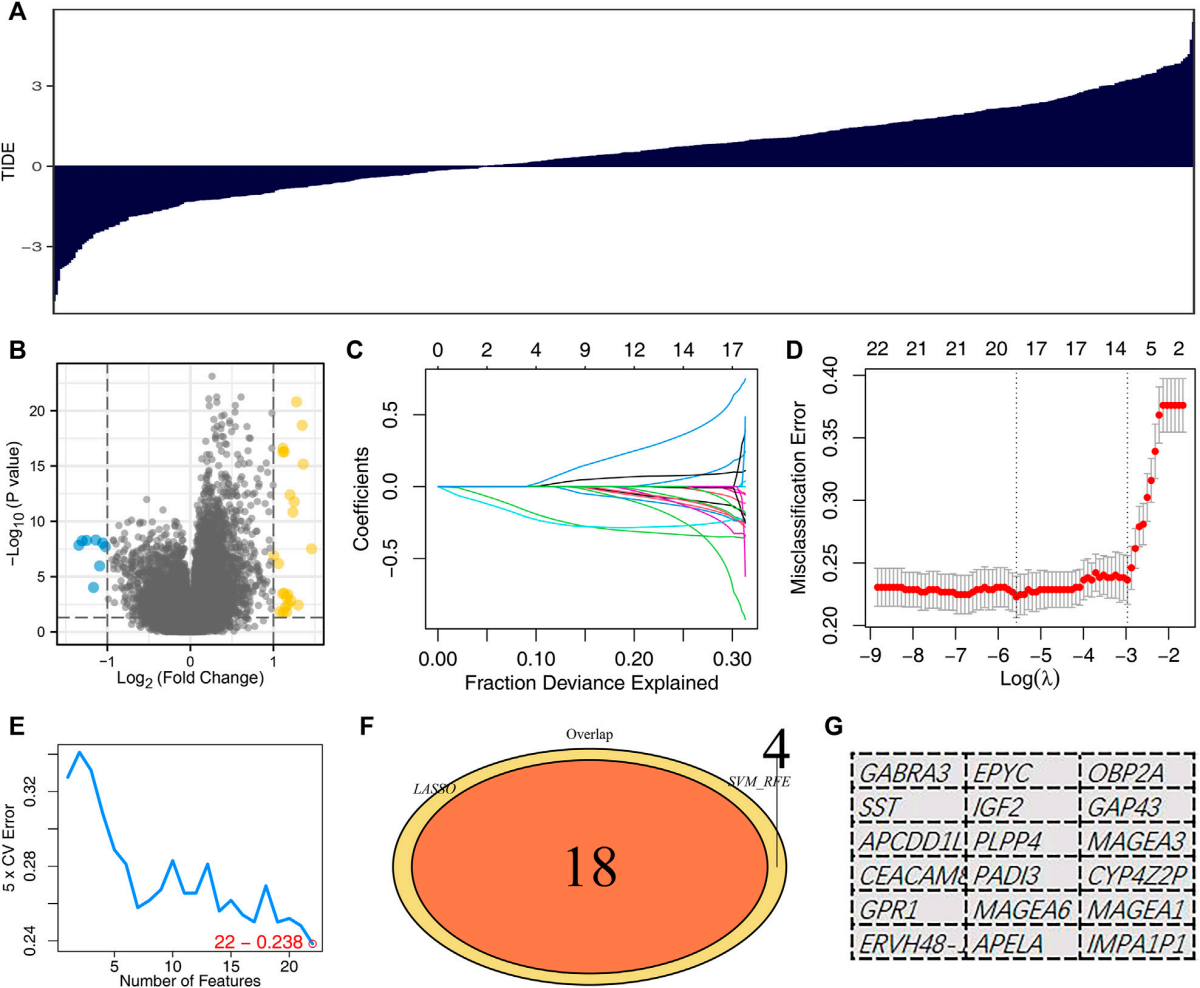
Identification of the immunotherapy characteristic genes. Notes: **(A)** TIDE analysis was performed to evaluate the immunotherapy response of each patient. **(B)** Volcano plots depicted differentially expressed genes between immunotherapy responders and non-responders; (**C,D)**: LASSO logistic regression analysis based on differentially expressed genes between immunotherapy responders and non-responders. **(E)** SVM-RFE analysis based on differentially expressed genes between immunotherapy responders and non-responders; (**F,G)**: The intersection of LASSO logistics regression and SVM-RFE algorithms identified 18 immunotherapy characteristic genes.

The whole flow chart of this study was shown in Supplementary Figure S1. To obtain immune response-related genes, we first divided patients into the immunotherapy responders group and the non-responders group. Figure 1A showed the TIDE score of each LUAD patient. The patients with TIDE score <0 were defined as the immunotherapy responder group, and those with TIDE score >0 were the non-responder group. The “limma” package was utilized to screen for differential expression analysis between the two specific groups, defined as immunotherapy-related candidate genes (Figure 1B). The LASSO logistic regression and SVM-RFE algorithm were used to optimize variable selection. The lung adenocarcinoma immunotherapy-related genes were screened by LASSO logistic regression and SVM-RFE, respectively. The LASSO logistic regression algorithm obtained a total of 18 immunotherapy characteristic genes (Figures 1C,D). The SVM-RFE algorithm screened 22 immunotherapy characteristic genes (Figure 1E). Lastly, the intersection of LASSO logistics regression and SVM-RFE algorithms identified 18 immunotherapy characteristic genes, including GABRA3, SST, APCDD1L, CEACAM8, GPR1, ERVH48-1, EPYC, IGF2, PLPP4, PADI3, MAGEA6, APELA, OBP2A, GAP43, MAGEA3, CYP4Z2P, MAGEA1, IMPA1P1 (Figures 1F,G).

### Predictive performance of immunotherapy response-related molecules

**FIGURE 2:**
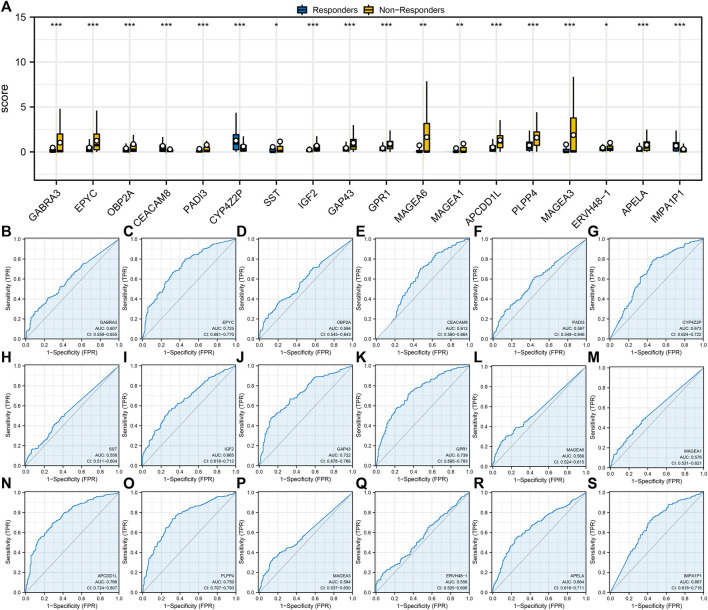
Evaluation of the prediction performance of immunotherapy characteristic genes. Notes: **(A)** Expression level of identified 18 characteristic genes in immunotherapy responders and non-responders; **(B–S)**: Prediction performance of 18 characteristic genes in LUAD immunotherapy response.

Next, we found a different expression pattern in characteristic genes between the immunotherapy responders and non-responders groups. For example, GABRA3, EPYC, OBP2A, PAI3, SST, IGF2, GAP43, GPR1, MAGA6, MAGEA1, APCDD1L, PLPP4, MAGEA3, ERVH48-1, and APELA were highly expressed in the immunotherapy non-response group, while CEACAM8, CYP4Z2P, and IMPA1P1 were highly expressed in the immunotherapy responders group (Figure 2A). In addition, ROC curves were utilized to assess the predictive performance of 18 characteristic genes for immunotherapy of LUAD. The AUC values of all characteristic genes were >0.5 and the AUC value of APCDD1L was 0.766 (Figures 2B–S).

### Construction of logistics model

To effectively evaluate the clinical efficacy of immunotherapy for each patient, we constructed the logistics model based on immunotherapy characteristic genes. Each patient was assigned a logistics score according to the formula: Logistics score = 1.14,241,470 + GABRA3 * -0.20857125 + EPYC * -0.28555130 + OBP2A * -0.23632246 + CEACAM8 * 0.20820226 + PADI3 * -0.04657940 + CYP4Z29 * 0.09164072 + SST * -0.03457475 + IGF2 * -0.92090211 + GAP43 * -0.24484709 + GPR1 * -0.32626723 + MAGEA6 * 0.36074564 + MAGEA1 * - 0.13692973 + APCDD1L * -0.3694431 + MAGEA3 * -0.29105109 + ERVH48-1 * -0.25158873 + APELA * -0.17219285 + IMPA1P1 * 0.75190815”.

**FIGURE 3:**
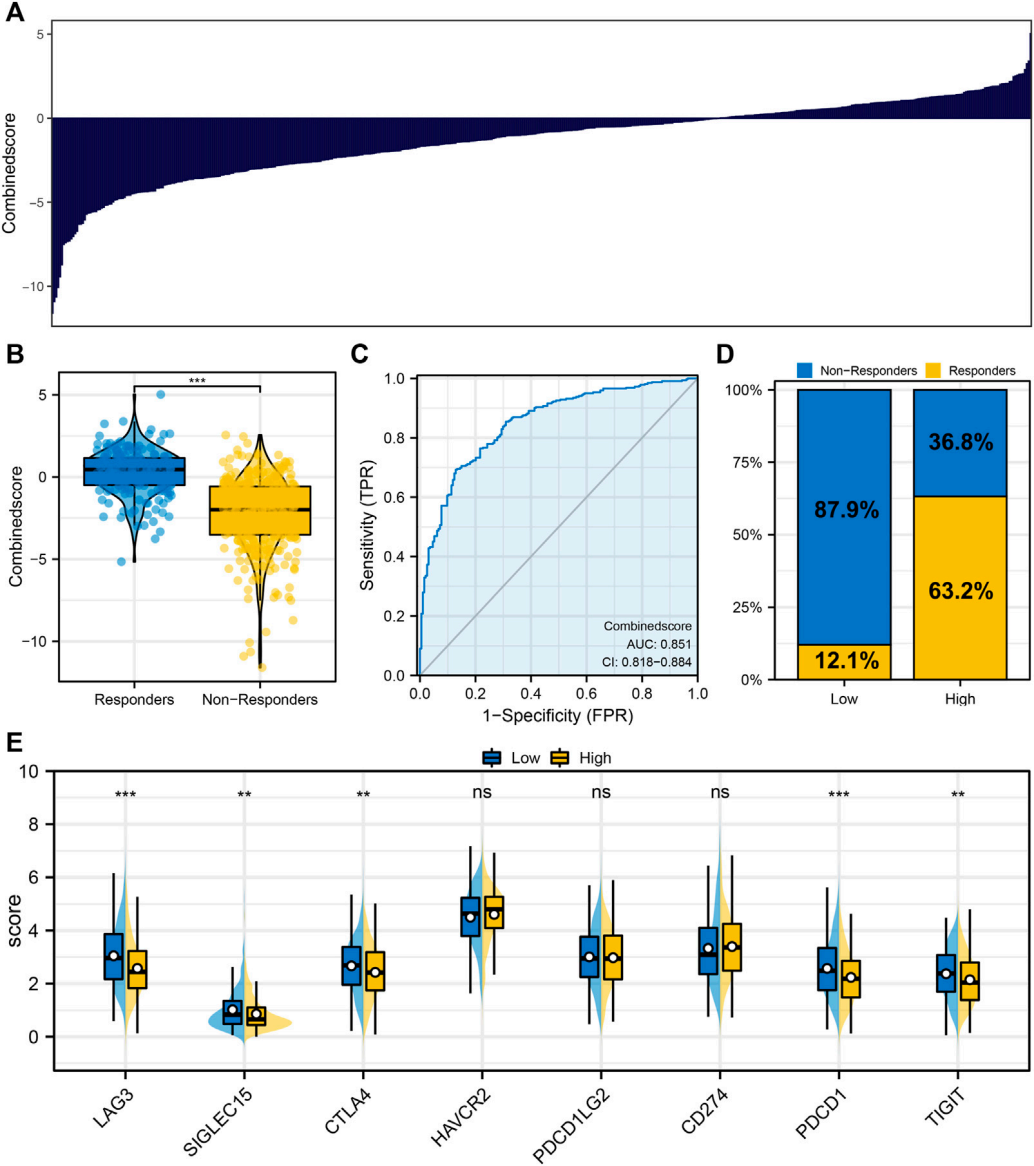
Logistics regression model. Notes: **(A)** Logistic regression model was constructed based on the identified characteristic genes; **(B)** Differences of logistics score between immunotherapy responders and nonresponders; **(C)** ROC curve was utilized to assess the performance of Logistics score in predicting the response to immunotherapy in LUAD patients; **(D)** Proportion of immunotherapy responders and non-responders in patients with high and low logistics score; **(E)** The level of key immune checkpoints in patients with high and low logistics score.

Figure 3A displayed the logistics score of each patient. We found that the patients in the immunotherapy responder group had a higher logistics score (Figure 3B). The ROC curve indicated that the logistics score had excellent performance in predicting LUAD immunotherapy response (Figure 3C, AUC: 0.851). Among the patients with low logistics scores, only 12.1% responded to immunotherapy significantly, while 63.2% of those with high logistics scores responded significantly (Figure 3D). In addition, we found a significantly higher level of CTLA4 and PDCD1 in patients with low logistics score (Figure 3E).

### Biological enrichment analysis

**FIGURE 4:**
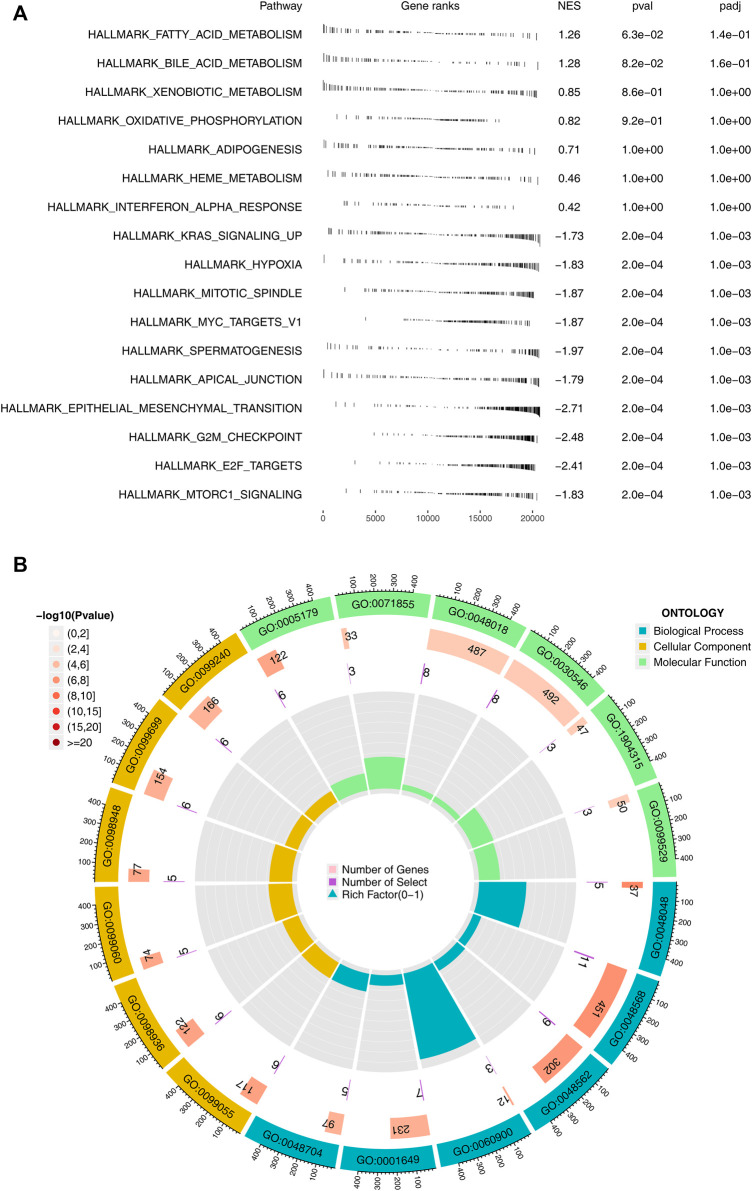
Biological enrichment analysis. Notes: **(A)** GSEA analysis between high and low logistics score based on the Hallmark gene set; **(B)** GO analysis between high and low logistics score.

Subsequently, we performed GSEA pathway analysis and GO analysis on patients with high and low logistics score subgroups. We found that pathways related to immunotherapy were mainly enriched in many metabolic pathways such as fatty acid metabolism, bile acid metabolism, oxidative phosphorylation, and carcinogenic pathways such as KRAS signaling up, MYC targets, epithelial-mesenchymal transition (Figure 4A). The loop diagram showed the enrichment of immunotherapy response-related genes in the pathway, such as GO: 0048568 and GO: 0048562 (Figure 4B). Kyoto Encyclopedia of Genes and Genomes (KEGG) based on GSEA analysis indicated that the terms of linoleic acid metabolism, arachidonic acid metabolism, ether lppid metabolism, alpha linolemic acid metabolism, fc epsilon ri signaling pathway, metabolism of xenobiotics by cytochrome P450, long term depression, glycerophospholipid metabolism, tyrosine metabolism were significantly enriched in the patients with high logistics score (Supplementary Figure S2).

### Tumor microenvironment assessment

**FIGURE 5:**
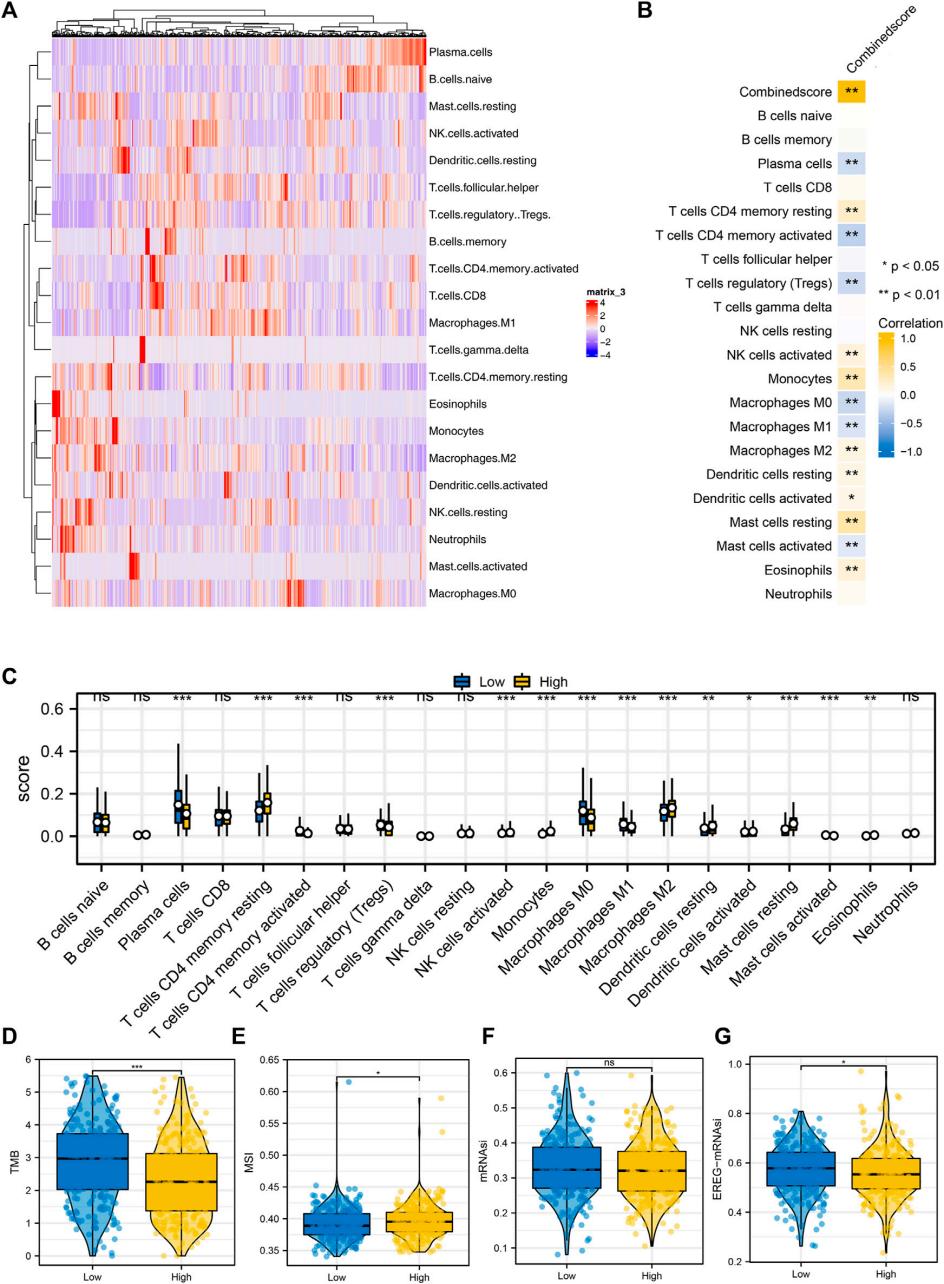
Immune infiltration analysis. Notes: **(A)** The CIBERSORT algorithm was utilized to quantify the immune infiltration in the LUAD tumor microenvironment; **(B)** Correlation of logistics score and quantified immune cells; **(C)** Infiltration level of quantified immune cells in patients with high and low logistics score; **(D–G)**: Level of TMB, MSI, mRNAsi and EREG-mRNAsi in patients with high and low logistics score.

The quantified immune cell in the tumor microenvironment of each LUAD patient was shown in Figure 5A. Correlation analysis showed that logistics score was positively correlated with NK cells activated, monocytes, macrophages M2, dendritic cells activated, and eosinophils, while negatively related to T cells CD4 memory activated, Tregs and mast cells activated (Figure 5B). Subsequently, the infiltration level of 22 immune cells in the high and low logistics score subgroups were shown in Figure 5C. Also, correlation analysis showed that TMB index and EREG-mRNAsi scores were higher in the low logistics score group, while MSI scored was higher in the high logistics score patients (Figures 5D–G).

### Role of ERVH48-1 in lung adenocarcinoma

**FIGURE 6:**
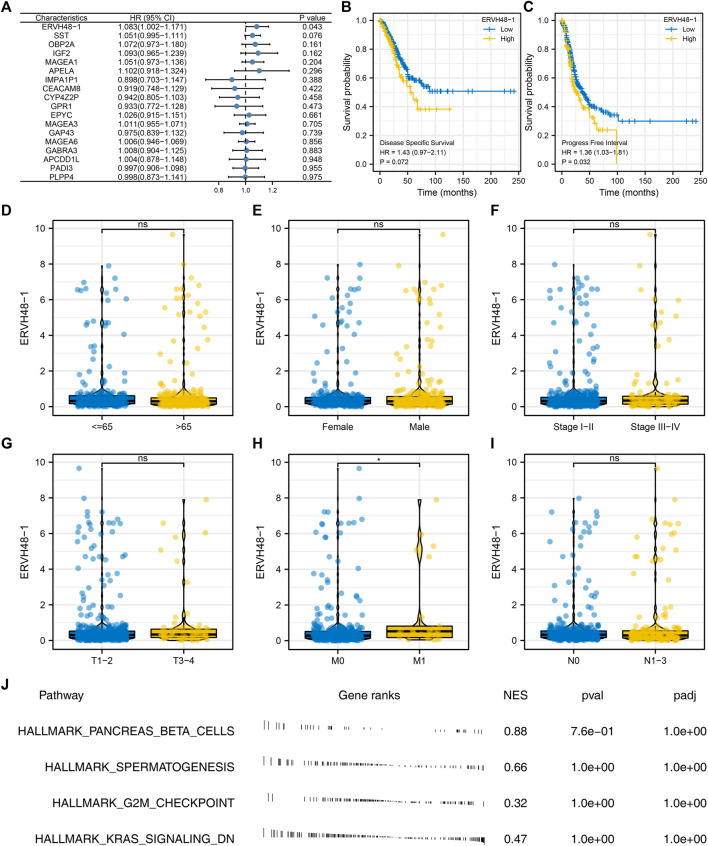
Further exploration of ERVH48-1. Notes: **(A)** Univariate Cox regression analysis of the characteristic genes; (**B,C)**: Difference in disease-specific survival and progression-free survival in patients with high and low logistics score; (**D–I)**: The expression of ERVH48-1 in populations with different clinical characteristics; **(J)** Pathway enrichment analysis of ERVH48-1.

Univariate Cox regression analysis suggested that ERVH48-1 was significantly associated with the clinical performance of LUAD patients, suggesting that this gene could affect both the immune response and the progression of LUAD (Figure 6A). Kaplan-Meier survival curves also indicated the significant effect of ERVH48-1 on patients disease-specific survival and progression-free survival (Figures 6B,C). Subsequently, the expression of ERVH48-1 in patients with different clinical features was detected, and it was found that the expression of ERVH48-1 was increased in patients with distant metastasis (Figures 6D–I). We performed pathway enrichment analysis and found that ERVH48-1 mainly changed related pathways such as pancreas beta cells, spermatogenesis, G2M checkpoint and KRAS signaling (Figure 6J).

**FIGURE 7:**
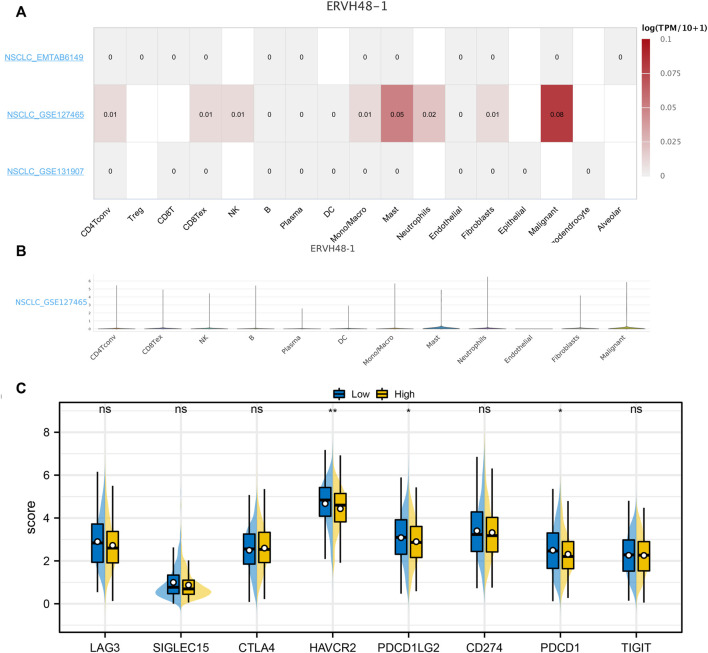
Single-cell analysis of ERVH48-1. Notes: **(A,B)**: The ERVH48-1 expression in different cell subgroups; **(C)**: The level of key immune checkpoints in patients with high and low logistics score.

Based on the online website, we evaluated the expression levels of ERVH48-1 in different cells. ERHV48-1 was mainly expressed in tumor cells and mast cells (Figures 7A,B). Moreover, we found that the expression of key immune checkpoints was decreased in the patients with high ERHV48-1 expression (Figure 7C). Results of qRT-PCR showed that ERVH48-1 was upregulated in lung cancer cells (Supplementary Figure S3).

## Discussion

In our investigation, we collected the TCGA-LUAD dataset to construct prognostic immunotherapy response features. The signature consisted of 18 immune response-related genes. Fain et al. observed that the downstream promoter in tumor cells overlaps with the DNA methylation site, and then activates the hypermethylation of its long transcript, resulting in a similar regional DNA hypermethylation pattern, including tumor suppressor genes (Fain et al., 2021). Another investigation demonstrated that the expression of PLPP4 in lung cancer patients with a higher malignant degree was also increased (Zhang et al., 2017). In our paper, a model consisting of 18 immune response characteristic genes can efficiently predict the clinical response of LUAD patients to immunotherapy.

In general, previous prognostic stratification prediction models are based on the real characteristics of tumors, like clinical TNM stage, tumor texture, vascular growth distribution and nerve infiltration. Certain components of innate and adaptive immunity were also actively involved in the progression of cancer (Rolfo et al., 2021). An investigation has shown that the immunological profiles (type, location and number of tumor-infiltrating immune cells) were a better predictor of patient survival than traditional histopathology in colon cancer (Dejima et al., 2021). The “Immunoscore” was a quantitative tool for predicting tumor immunogenicity. In addition to the current histopathological staging system, it is undergoing clinical research on a variety of cancer types (Pfirschke et al., 2016; Della Corte et al., 2020). In addition to determining tumor immune microenvironment from RNA sequencing data, immune characteristics can be used to predict patient clinical performance. In addition to the survival rate of patients, this immune characteristic was also a predictor of response to ICI treatment. In our investigation, patients with low logistics score had poor responses to immunotherapy, suggesting that for the selection of patients before ICI treatment, these 18 immune response-related characteristic genes may be useful. In the process of selecting patients for ICI treatment, PD-L1 expression, TMB, mRNAsi, and EREG-mRNAsi have been measured (Li et al., 2021a). The predictive performance of this immune feature was not related to mRNAsi. On the contrary, we found a significant decrease in TMB in the high logistics score group. Due to logistics score being a complex model with multiple variables, we believe that other variables may help to improve the prediction effect of logistics score groups.

Based on the cancer-immune cycle hypothesis, the anti-tumor effect involved many gradual processes (Duhen et al., 2018). Cancer develops when some steps of the process are hindered, including an increase in immune checkpoint expression, impaired T-cell infiltration, and antigen regulation (Li et al., 2021b). As a result, patients may benefit little from ICI treatment when immune checkpoints are not the only rate-limiting step. In our investigation, patients with high logistics score had higher immune checkpoint molecules level. It can be inferred from the higher levels of immune checkpoint molecules that the high logistics score group already possessed T cell activation. Therefore, patients with high logistics scores may be more sensitive to ICI treatment. In the clinical, the application of our logistics model might contribute to the therapy section of the individual patient.

Although some positive results have been achieved, there were still some limitations. Firstly, this immune feature was constructed based on public data sets. The predictive ability needs to be further verified in randomized controlled cohorts. In addition, we used the logistics score to simulate the patients response to ICI treatment. But there are not enough immunotherapy cohorts to validate our model, so the logistics score still cannot completely replace the real treatment response.

## Data Availability

Publicly available datasets were analyzed in this study. This data can be found here: https://portal.gdc.cancer.gov/.
