# Supplementary figures and images for: Immunotherapy response and microenvironment provide biomarkers of immunotherapy options for patients with lung adenocarcinoma
Source: Front Genet. 2022 Oct 25;13:1047435. doi: 10.3389/fgene.2022.1047435 (PMC9640754; doi:10.3389/fgene.2022.1047435)

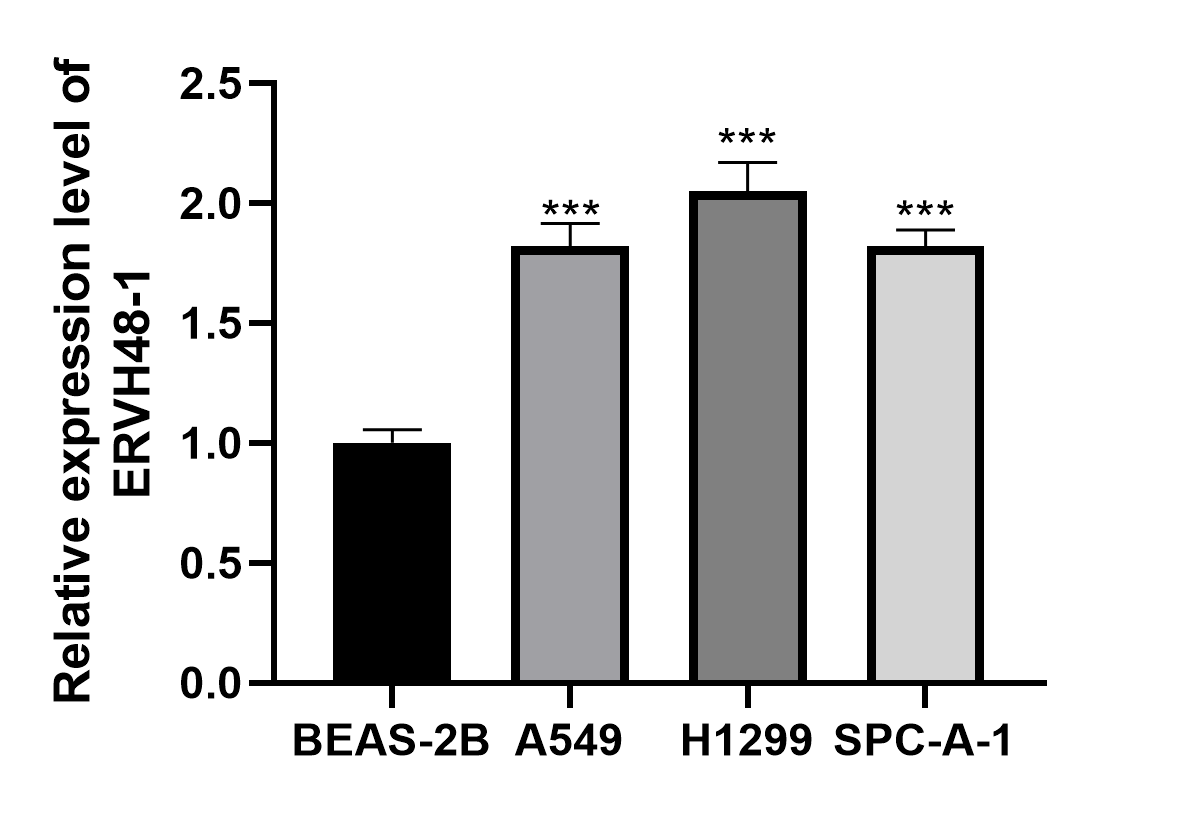

Supplement: Supplementary file 1 [file Image3.TIF]

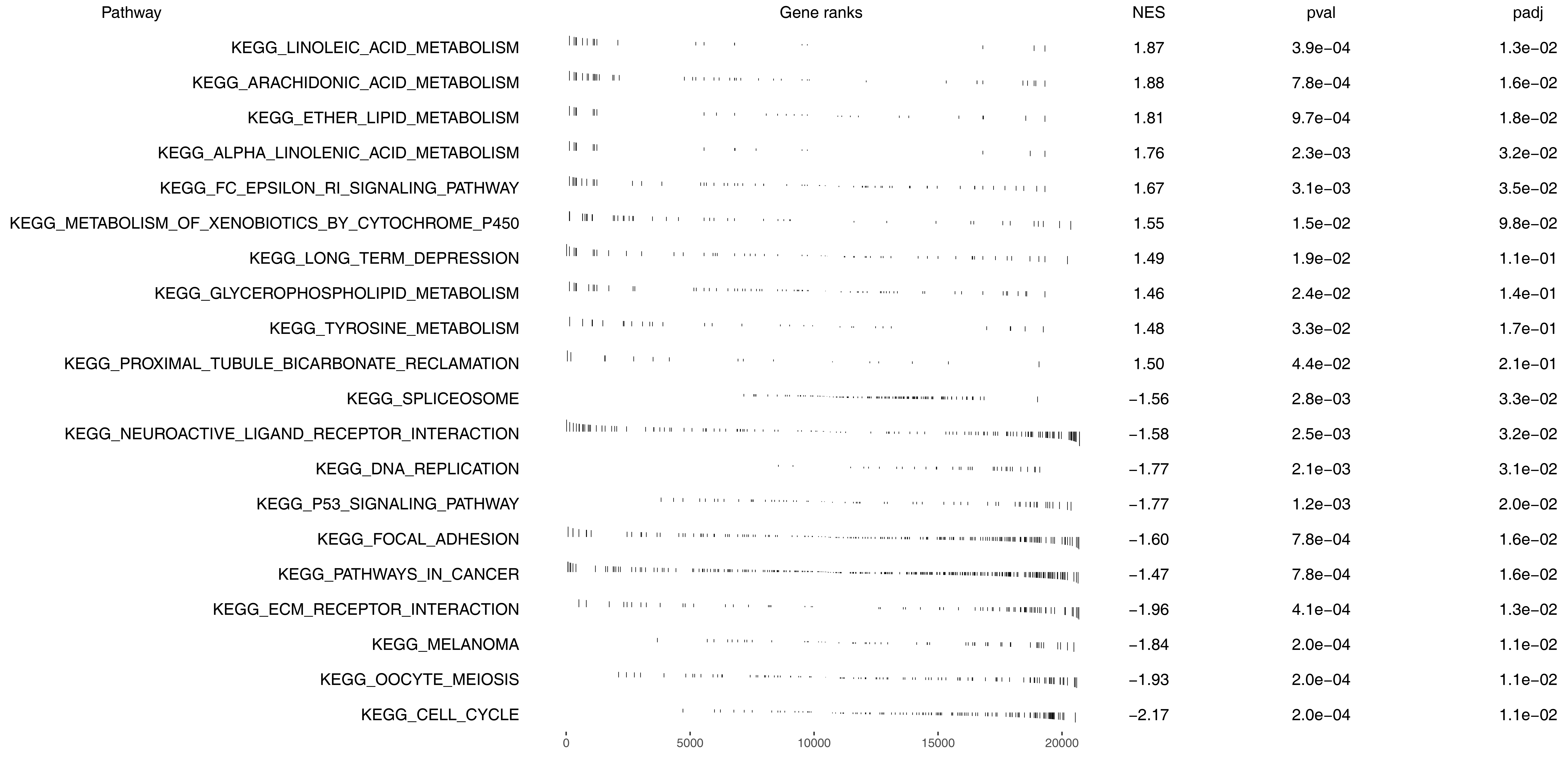

Supplement: Supplementary file 2 [file Image2.TIF]

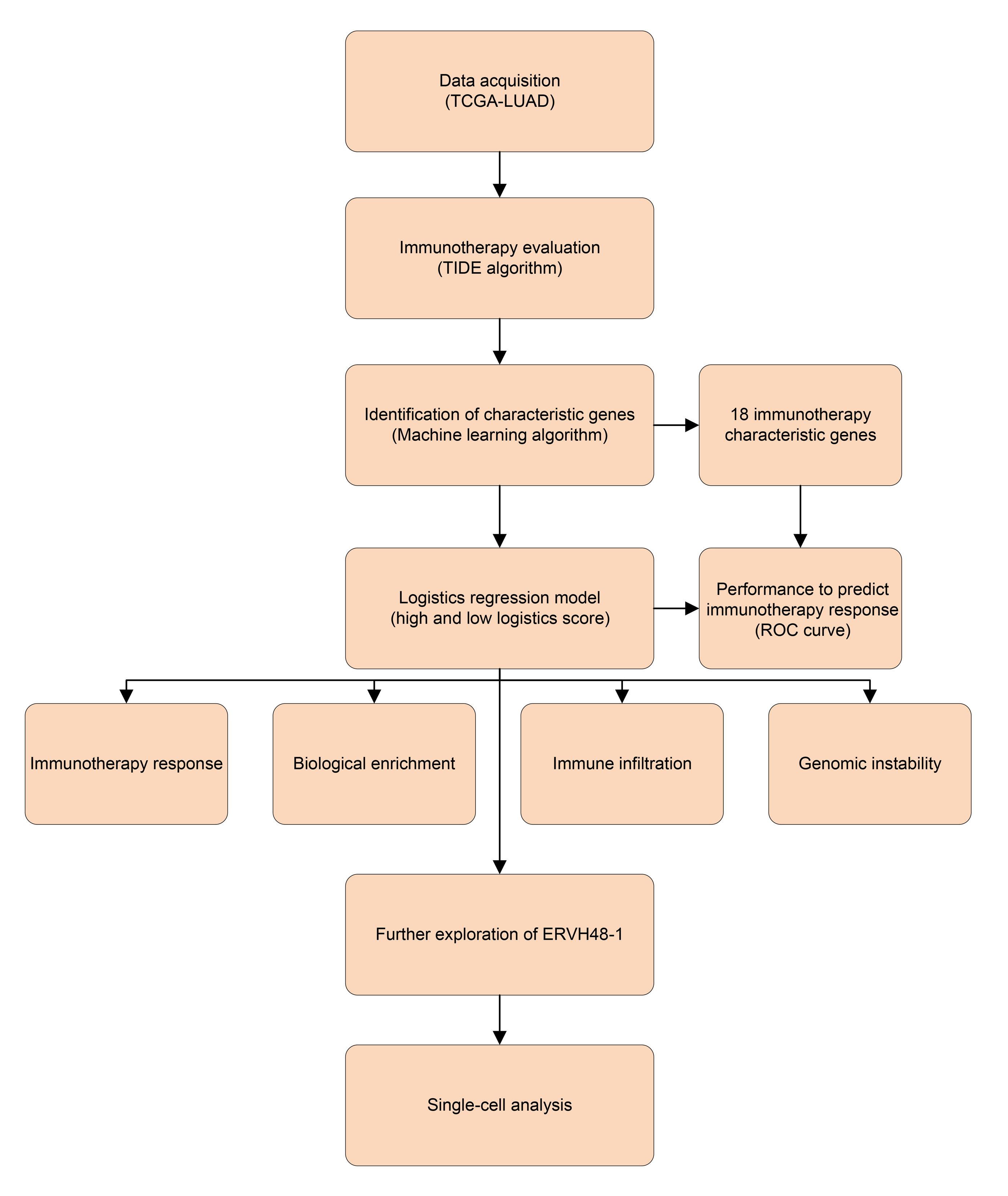

Supplement: Supplementary file 3 [file Image1.TIF]
